# Avian influenza viruses that cause highly virulent infections in humans exhibit distinct replicative properties in contrast to human H1N1 viruses

**DOI:** 10.1038/srep24154

**Published:** 2016-04-15

**Authors:** Philippe F. Simon, Marc-Antoine de La Vega, Éric Paradis, Emelissa Mendoza, Kevin M. Coombs, Darwyn Kobasa, Catherine A. A. Beauchemin

**Affiliations:** 1 Department of Medical Microbiology, University of Manitoba, Winnipeg, MB Canada; 2 Special Pathogen Program, National Microbiology Laboratory, Public Health Agency of Canada, Winnipeg, MB Canada; 3 Department of Immunology, University of Manitoba, Winnipeg, MB Canada; 4 Department of Physics, Ryerson University, Toronto, ON; 5 Manitoba Centre for Proteomics and Systems Biology, Winnipeg, MB Canada

## Abstract

Avian influenza viruses present an emerging epidemiological concern as some strains of H5N1 avian influenza can cause severe infections in humans with lethality rates of up to 60%. These have been in circulation since 1997 and recently a novel H7N9-subtyped virus has been causing epizootics in China with lethality rates around 20%. To better understand the replication kinetics of these viruses, we combined several extensive viral kinetics experiments with mathematical modelling of *in vitro* infections in human A549 cells. We extracted fundamental replication parameters revealing that, while both the H5N1 and H7N9 viruses replicate faster and to higher titers than two low-pathogenicity H1N1 strains, they accomplish this via different mechanisms. While the H7N9 virions exhibit a faster rate of infection, the H5N1 virions are produced at a higher rate. Of the two H1N1 strains studied, the 2009 pandemic H1N1 strain exhibits the longest eclipse phase, possibly indicative of a less effective neuraminidase activity, but causes infection more rapidly than the seasonal strain. This explains, in part, the pandemic strain’s generally slower growth kinetics and permissiveness to accept mutations causing neuraminidase inhibitor resistance without significant loss in fitness. Our results highlight differential growth properties of H1N1, H5N1 and H7N9 influenza viruses.

Influenza A viruses (IAV) are a constant threat to public health and the cause of annual epidemics as well as global pandemics^1^,^2^. Global mortality due to seasonal IAV infections is generally estimated at approximately 500,000 deaths in over 3,000,000 acute cases every year^1^,^3^. Not all IAV cause the same clinical manifestations. In healthy adults, seasonal flu — although highly contagious — is usually a self-limiting disease causing fever, myalgia, malaise and cough^4^. The H1N1 and H3N2 influenza A viruses that are responsible for seasonal influenza in humans have been in circulation since 1977 and 1968, respectively^5^. In 2009, a novel strain of the H1N1 subtype caused an influenza pandemic, and this strain has since become established as a seasonal strain, largely replacing previous H1N1 strains from circulation in the 2014–2015 influenza season^6^,^7^.

The animal reservoir for most subtypes of IAV are wild aquatic birds, encompassing viruses of the 16 HA and 9 NA subtypes^8^,^9^, whereas bats have recently been shown to harbour H17N10 and H18N11 subtyped IAVs^10^. Avian influenza viruses are at the origin of infections in a wide range of avian and mammalian species. Some of these strains which circulate in birds have also caused limited, but serious human infections^11^. Despite their high lethality, these strains typically do not show significant human-to-human transmission^11^. Most prominent is the highly pathogenic avian influenza (HPAI) H5N1 subtype virus that was first identified in human cases of infection in 1997^12^. Since then, this subtype has been in circulation in migratory birds and has caused large-scale poultry outbreaks in Asia, Europe and Africa as well as infection in nearly 700 human cases with about 60% lethality^11^. In January 2014, a traveler returning from China died of an H5N1 infection in a Canadian hospital. This was the first human case of avian influenza in North America^13^. In March 2013, an H7N9 virus emerged from birds in China and had, as of February 2015, infected over 500 people with a 20% lethality rate^14^,^15^,^16^. As with the H5N1 virus, two imported human cases of H7N9 have been documented in North America^17^. Clinically, the course of infection with H7N9 strains in humans is similar to that observed for infections with H5N1 strains (reviewed in ref. 18), but the H7N9 virus lacks the polybasic cleavage site in its hemaggalutinin surface protein, a hallmark and well-described molecular marker of HPAI viruses^19^. Interestingly, while the H7N9 virus causes HPAI-like disease in humans, it is a low pathogenicity avian influenza (LPAI) virus as it causes only mild illness in domestic poultry^18^. The continued spread of those avian IAV in migratory birds and ongoing sporadic infections in human and commercial poultry continues to pose a significant public health risk both for localized outbreaks and global pandemics.

A great deal of investigation has been dedicated to the identification and delineation of the role of viral determinants of pathogenicity and adaptation of virus to new hosts following zoonotic transmission of viruses, including, for example, the identification of HA as the major virulence determinant in 1918 H1N1 Spanish influenza strain^20^ or the aerosol transmission of H5N1 HPAI in ferrets^21^,^22^. The consequences of these viral determinants of pathogenicity are frequently characterized by comparison of viruses containing these determinants with low virulence variants in animal models and by *in vitro* growth kinetics (e.g.^23^,^24^,^25^,^26^,^27^,^28^,^29^,^30^,^31^). Strains are characterized on the basis of properties that are accessible to assay with existing methodologies such as receptor affinity of the viral receptor binding protein hemagglutinin (HA)^27^, enzymatic activity of neuraminidase (NA) or transcriptional activity of the viral polymerase. However, most methods characterize discrete properties or activities of viral proteins or define the properties of a virus by readouts, such as virus yield *in vitro* or disease outcome *in vivo*, without providing any information regarding the dynamics of interaction between a virus and its host cell.

Combining experimental viral kinetic results — such as yield assays measured by 50% Tissue Culture Infectious Dose (TCID_50_), plaque assay, and/or qRT-PCR — with mathematical modelling is a powerful approach to characterize the details of viral replication *in vitro*^32^,^33^,^34^,^35^,^36^,^37^ and in other applications^38^,^39^,^40^,^41^,^42^. Generally, mathematical modelling of viral kinetics involves fitting a set of ordinary differential equations (ODEs) to experimental data^39^. The ODEs provide a mathematical framework that describes the variation of viral concentration as a function of time. This in turn enables the identification of the individual steps of the virus replication cycle; such as the time of initial infection, the latent or eclipse phase, the viral production phase, and finally the decay and death of the cell. Examples of a viral strain’s kinetic parameters that can be determined include the duration of the eclipse phase (i.e. the time elapsed between successful cell infection and the start of virus progeny release by that cell (*τ*_*E*_)); the duration of the infectious phase (i.e. the period following the eclipse phase during which an infected cell is infectious (*τ*_*I*_)); the virus production rate throughout the infectious phase (*p*_*RNA*_ or *p*_*TCID*50_), and the virus degradation or loss of infectivity rate (*c*_*RNA*_ or *C*_*TCID*50_). Extracting these kinetic parameters allows for a greater understanding of the mechanics behind an individual strain’s replicative capacity^43^. For example, having a markedly short eclipse phase might prove to bear a competitive advantage. Mathematical models make it possible to compare and quantify the role of these parameters in the replication of a variety of IAVs^32^,^44^,^45^,^46^. Importantly, the RNA measured here by qRT-PCR is the total viral RNA found in supernatants and is not the intracellular or cRNA found in cells. This, combined with infectious particle count in the form of TCID_50_ forms the basis of estimating the amount of infectious virions released in the supernatant. Using this approach, we have previously showed that the H275Y mutation, associated with resistance to NA inhibitor, in both a seasonal H1N1 strain^44^, as well as a 2009 pandemic H1N1 strain^45^, affects the virus life cycle by increasing the eclipse phase while affecting the burst size. This enhanced our understanding of the mechanisms driving viral fitness and the complete dominance of the H275Y mutation — a marker of resistance to antiviral NA inhibitor drugs — in circulating strains prior to 2009 even in the absence of selective antiviral pressure^47^,^48^,^49^. A major strength of this approach is its ability to translate *in vitro* results into information on the fundamental replicative capacity of virus that are also applicable *in vivo*^37^,^45^. A detailed understanding of each aspect of viral replication is important not only for fundamental virology but also for the design of novel therapeutic strategies as it allows a better understanding of which steps of the viral replication can be targeted for maximum inhibition.

Here, using a mathematical model of infection^50^, we have analyzed influenza viruses causing a continuum of illness, from mild, seasonal infections to HPAI virus infections that cause significant morbidity and mortality in humans. The results provide a detailed quantification of the replication parameters characterizing influenza infections with seasonal H1N1, 2009 pandemic H1N1, LPAI H7N9 and HPAI H5N1 viruses, and highlight the distinct differences in their replicative strategies, despite the fact that they all adhere to the same basic processes for replication.

## Results

**Figure 1:**
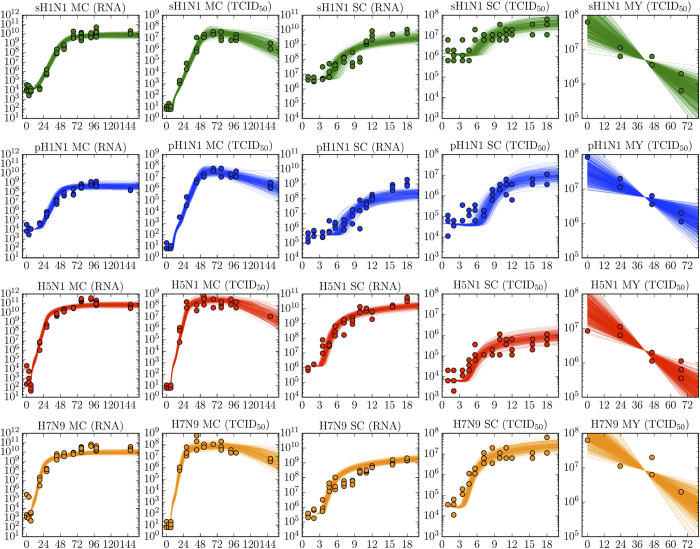
Comparison of infection kinetics and agreement with the mathematical model. The experimental data for the low-MOI, multiple-cycle (MC), the high-MOI, single-cycle (SC) infection assay, and the mock-yield (MY) assay are represented as circles while the lines represent the time courses of 900 *in silico* infections simulated using our mathematical model and the parameters selected by our MCMC process. At each time point, supernatant samples were harvested in triplicate, titrated by TCID_50_, and viral RNA was quantified by qRT-PCR. Color coding as follows: Seasonal H1N1 (sH1N1, green); 2009 pandemic H1N1 (pH1N1, blue); H5N1 (red) and H7N9 (orange). “Y” axes are either TCID50/ml or RNA copies/ml and “X” axes are time post-infection in hours. These data were used to extract the probability distributions for the parameters characterizing the replication efficacy of each virus (Fig. 2, Table 1).

We evaluated and compared the infection kinetics of two strains of human influenza A H1N1 causing relatively mild disease in humans, a seasonal A/New Caledonia/20/1999-like clinical isolate (hereafter sH1N1) and the 2009 pandemic H1N1 strain A/Mexico/INDRE4487/2009 (hereafter pH1N1), as well as two viruses causing severe human infections, the HPAI H5N1 strain A/Indonesia/05/2005 (hereafter H5N1) and the LPAI H7N9 strain A/Anhui/1/2013 (hereafter H7N9). For this purpose, two distinct infection assays were performed which, together, provide complementary information on different aspects of the replication kinetics of these strains. The single-cycle (SC) assay is done with infection at high MOI (3 PFU/cell) such that all cells are infected approximately simultaneously. The viral load produced over time allows direct observation of the average timing for initiation of virus release (the duration of the eclipse phase) and the kinetics of virus production within a single, newly infected cell. The multiple-cycle (MC) assay is done with infection at low MOI (0.01 PFU/cell) where a small population of initially infected cells provide the viral progeny necessary to trigger a second cycle of infection. This causes second and third cycles allowing one to observe the average period and amplitude of successive infection cycles. A cell-free, mock-yield (MY) assay was also performed to evaluate the stability of virus infectivity over time at 37 °C. The total viral RNA (copies/mL) and infectious (TCID_50_/mL) virus concentration found over time in the supernatant of these three assays for each of the four viruses are presented in Fig. 1.

Overall, the most striking differences are observed in the MC infections for the peak titers. The H5N1 and H7N9 viruses reached higher maximum average titres (H5N1 = 3.67 × 10^8^ TCID_50_/ml; H7N9 = 2.89 × 10^8^ TCID_50_/ml; sH1N1 = 2.52 × 10^7^ TCID_50_/ml; pH1N1 = 4.48 × 10^7^ TCID_50_/ml), and reached them more rapidly, at 42h and 53h p.i. for the H5N1 and H7N9 viruses, and at 66h p.i. for the sH1N1 and pH1N1 strains. This suggests that the H5N1 and H7N9 viruses undergo successive infection cycles more rapidly than the sH1N1 and pH1N1 strains and/or infect a greater number of cells within each cycle. In the SC infection assay, intensive virus production and release also appears to begin earlier for the H5N1 and H7N9 viruses, beginning around 3h–4h p.i. compared to 5h–6h for the sH1N1 and pH1N1 strains, suggesting a longer delay for virus production and release for the two H1N1 strains, consistent with longer elapsed time between successive infection cycles with these strains.

**Figure 2:**
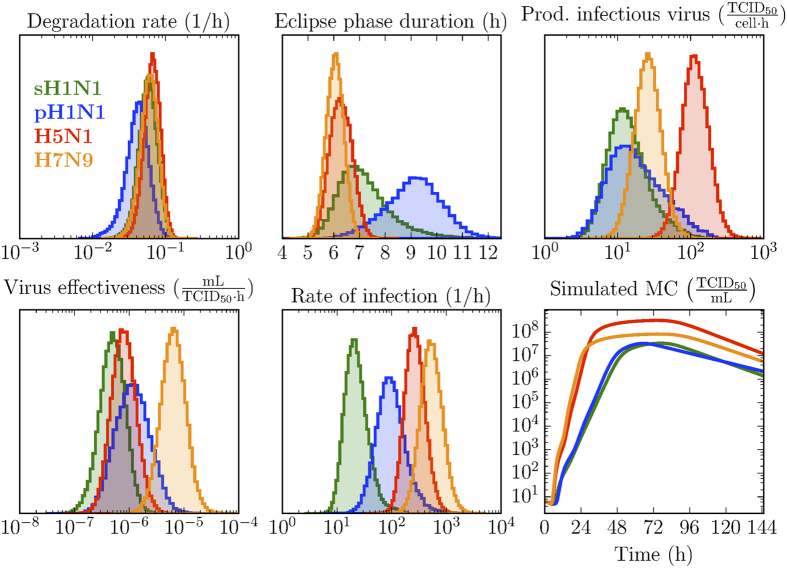
Parameters characterizing the viral replication efficiency. Probability density functions of the key parameters for each of the four strains. The degradation rate is a measure of the stability of the virions’ infectiousness. The eclipse phase is the time elapsed between the successful infection of a cell and the release of its first virion. Once virus production and release is well underway in the infected cell, infectious virus progeny will be produced at a certain rate (Prod. infectious virus, measured in TCID_50_/cell/h). The effectiveness of the infectious progeny is quantified separately as the rate at which new cells are infected per TCID_50_ of progeny. Together, the combined effect of the rate of production and the effectiveness of the infectious progeny will result in a number of infections per hour (Rate of infection, 1/h). The simulated multiple-cycle (MC) assay (bottom-right panel) was produced by using the best-fit parameters obtained with our MCMC method and illustrates how these parameters, together, yield the observed viral growth kinetics.

To gain a quantitative understanding of these differences, the experimental data were analyzed using our mathematical model, and a Markov chain Monte Carlo (MCMC) method was used to determine the likelihood distribution of the parameters characterizing the replication efficiency of each of the four viruses. Figure 1 shows 900 mathematically-simulated infection time courses for each of our four influenza viruses, along with the experimental data collected for these same infections performed *in vitro*. These 900 different infection time courses were simulated using 900 different parameter sets corresponding to the last three steps of the 2,000 MCMC steps performed in each of the 300 parameter chains obtained for each strain (i.e., the final 900 out of 600,000 parameter sets). As such, this set of time courses illustrates how the uncertainty in the virus’ replication parameters due to experimental variability translates into deviations in the shape of the infection time course predicted by the mathematical model. The corresponding replication parameters obtained for each virus are reported in Table 1. Figure 2 presents the probability distributions for the value of key parameters characterizing different aspects of the replication efficiency of each virus.

For all viruses, the rate at which virions lose infectivity in the medium at 37 °C is similar. The duration of the eclipse phase — the time elapsed from the successful infection of a cell by a virion to the release of the first virion produced by that cell — is comparable for the H5N1 and H7N9 viruses. It is shorter (by ~1 h) than that of the sH1N1 strain, though this difference is not statistically significant. The H5N1 and H7N9 eclipse phases were found to be significantly shorter (by ~3 h) than that of the human pH1N1 strain. As the newly infected cells emerge from the eclipse phase, they begin virus production and release. Cells infected with the sH1N1, pH1N1, or H7N9 virus produce infectious virions at comparable rates (within 2-fold of one another), but those infected with the HPAI H5N1 virus produce significantly (4–9 times) more, the most of the four viruses. Once the virions are produced, they go on to infect other cells. Per infectious unit (TCID_50_), the H7N9 virus has higher effectiveness, i.e. caused infection significantly faster, than all other viruses studied, namely 5, 8, and 12 times faster than the pH1N1, H5N1, and sH1N1 viruses, respectively. But once cells begin producing virus, the rate at which they cause the infection of other cells is a combination of the rate at which they produce infectious virus units and the speed or effectiveness with which these units can cause infection. The rate at which a productively infectious cell causes secondary infections — the combined effect of these 2 processes — is therefore a good measure of the overall speed of infection of a particular strain once virus production gets underway, i.e. after the initial eclipse phase. The overall rate of infection with the sH1N1 virus is the lowest, significantly less than the pH1N1 virus which causes infection 4 times faster, with the H5N1 and H7N9 viruses causing infection 3–6 times faster than the pH1N1 virus.

Interestingly, we find that the H5N1 and H7N9 viruses cause a comparable number of infections per hour, despite the significantly higher infectious production rate of H5N1 virions compared to H7N9 virions. This is due to the significantly higher effectiveness of the H7N9 virus at causing infection — 10 times faster per infectious unit — than the H5N1 virus. This could mean that there are significant differences in receptor recognition and affinity leading to more efficient infection by the H7N9 virus. The H5N1 and H7N9 viruses also cause the infection of 3–6 times more cells per hour than pH1N1, and 12–24 times more than sH1N1. This can be attributed to a higher rate of infectious virus production in the case of the H5N1 virus, and to each infectious virion more rapidly causing infection in the case of the H7N9 virus. Ultimately, the shorter eclipse phase and the higher rate of infections per hour observed for the H5N1 and H7N9 viruses all contribute to their significantly more rapid (by ~1 day) infection progression (the up-slope of the viral titer curves) and higher peak viral loads, compared to that seen for infections with the two H1N1 strains. In particular, we find that the virus which causes the mildest infection in humans, sH1N1, has the slowest rate of infection, significantly slower than that for the other three viruses evaluated. This is despite its virus progeny exhibiting the highest effectiveness at causing infection, likely because it produces infectious virions at the lowest rate.

## Discussion

Aside from bats harbouring H17N10 and H18N11 subtypes^10^, wild waterfowl are considered to be the main reservoir for IAV, harbouring all viruses with the 16 HA and 9 NA subtypes associated with influenza in birds and which are the source of influenza viruses that are transmitted to other animal species^8^,^9^. Comparatively, humans can be considered accidental hosts of IAV as only a handful of subtypes have been able to gain a foothold and establish themselves in the population. Seasonal human strains of H1N1, H3N2 and H2N2 subtypes have circulated since the pandemic of 1918^5^. Only a few HPAI strains (including subtypes H5N1, H7N7 and H7N3) and LPAI strains (which include but are not limited to subtypes H9N2 and H7N9) have thus far crossed the species barrier and infected humans^8^. Importantly, most human infections with avian strains result in only a mild if detectable disease similar to seasonal influenza. However, HPAI H5N1 and the recent LPAI H7N9 strains cause disease with acute symptoms and a high mortality rate^8^. Many factors need to be taken into account to explain the different degree of virulence of influenza strains. Host immunity and genetics, routes and doses of infection, viral tropism, replicative capacities of strains all combine to create infections ranging from mild to lethal. *In vitro* studies offer an isolated system where strain-specific differences in viral replication kinetics and associated, intracellular host processes can be studied independently of host factors such as immune responses, genetics, infection route and doses.

In a study of a 2009 pandemic H1N1 strain (pH1N1, strain California/04/2009 H1N1 – (CAL04)), Itoh *et al.*^26^, found that its growth peaked at 48h post-infection (p.i.) when grown at 35 ^o^C in Madin-Darby Canine Kidney (MDCK) cells infected at an MOI = 0.001. In MDCK and differentiated Normal Human Bronchial Epithelial (NHBE), whether at 33 ^o^C or 37 ^o^C, it appears that A/Anhui/1/2013 H7N9 (the same strain used in the present study) and CAL04 grew to similar titers and peaked at roughly the same times^27^. This is in contrast with our results where the H7N9 strain significantly outgrew the pH1N1 strain. The argument for growing the viruses at 33 ^o^C is that it better mimics human upper respiratory tracts while 37 ^o^C better mimics the lower respiratory tract. Therefore, a strain exhibiting high levels of replication at 33 ^o^C may infect humans more easily. In a study focusing on temperature-sensitive mutants used for live attenuated vaccine production, Broadbent *et al.*^51^ compared the growth of lab-adapted H1N1, 2009 pandemic H1N1 (pH1N1) and HPAI H5N1. Endpoint titres were measured after 72 h of incubation in MDCK cells infected at a MOI of 0.01 and incubated at temperatures ranging from 33 ^o^C to 42 ^o^C. The lab-adapted H1N1 strain exhibited optimal growth at 37 ^o^C with approximately one log reduction at 33 ^o^C. Conversely, the pH1N1 strain showed optimal titers at 33 ^o^C with one log reduction at 37 ^o^C. The H5N1 strain showed similar titers at both temperatures. Also in MDCK cells, Li *et al.*^52^ compared the growth kinetics of swine-origin H1N1 isolate (S-OIV), sH1N1 and H5N1 strains. They found that the optimal growth rates for all strains was at 37 ^o^C and that in cells incubated at 33 ^o^C the sH1N1 strain had slightly higher titres (approximately half-log) then the S-OIV and H5N1 strains although no statistics were provided. Still in MDCK cells infected at an MOI of 0.01, a pH1N1 virus showed similar growth rate and peak titres at both 33 and 37 ^o^C while an sH1N1 strain showed a marginal increase in titers at 37 ^o^C compared to 33 ^o^C^53^. Using Newborn Pig Trachea (NPTr) as well as MDCK cells infected at MOI of 0.001, growth of seven strains of H1N1, H1N2 and H3N2 belonging to different swine and avian lineages was compared^54^. While it appears that the replication at 33 ^o^C was delayed compared to 37 ^o^C, the growth rates were similar. However, the experiments were carried over only 48 h which may have masked the peak titres of some strains. Using primary human airway epithelial cells (HAE), growth kinetics of seasonal human H3N2, avian H5N3 and HPAI H5N1 strains were determined^55^. Over 72 h, the human viruses showed similar growth at both 32 ^o^C and 37 ^o^C, the avian H5N3 grew well at 37 ^o^C but did not grow at 32 ^o^C and the H5N1 HPAI strain exhibited reduced growth at 32 ^o^C compared to 37 ^o^C. In differentiated primary human bronchial and tracheal epithelial cells (HTE) cultured in transwells under air-liquid interface, infection with sH1N1, HPAI H5N1 and novel H7N9 strains exhibited significant differences between the strains when grown at 33 and 37 ^o^C^56^. The sH1N1 strain had identical growth at both temperatures while the H5N1 and, to a lesser extent the H7N9 showed reduced growth at 33 ^o^C. All the studies mentioned above used traditional TCID_50_ infectivity measure as their end-point and, in some cases^51^,^54^ qRT-PCR methods. Solely using qRT-PCR, Kasloff *et al.*^57^ assessed the replication of 11 IAV. These include isolates of HPAI H5N1, novel H7N9, sH1N1 and the 1918 pandemic H1N1 viruses. Immortalized swine pulmonary macrophage cells (IPAM) were infected at MOI = 0.07 and incubated at 33, 37 and 41 ^o^C. In that cell line, the avian viruses had a preference for the 37/41 ^o^C incubations, although only two of the five tested viruses showed statistically-significant differences in their copy numbers at 48h p.i. For the human viruses, only the 1918 pandemic strain had significantly better growth at 37 ^o^C compared to 33 ^o^C. Taken together the above results suggest that optimal temperature for most isolates is generally 37 ^o^C, with human viruses better able to replicate at the lower temperatures. In our study, we focused solely on replication at 37 ^o^C in human lung epithelial cells (A549) and comparing human (sH1N1 and pH1N1) and avian (HPAI H5N1 and novel H7N9) strains. Yamaji *et al.*^31^ studied the *in vitro* growth kinetics in A549 cells of seven strains of H5N1, isolated from human and birds. For cells infected at an MOI of 0.0002 and cultured at 37 ^o^C, peak titers where attained at roughly 48h post-infection. Differences were noted when cultivating the cells at 33 ^o^C or 37 ^o^C with human H5N1 viruses growing to higher titers at 33 ^o^C. A study by Zhang *et al.*^28^ showed that H5N1 viruses isolated after one or five passages in embryonated chicken eggs reached peak titers between 36 and 48 h in A549 infected at MOI of 0.001 and cultured at 37 ^o^C. There was a trend for slightly faster kinetics in the P5 viruses vs the P1 viruses. In another study, A549 cells infected with a seasonal H1N1 strain similar to the one used in our study (A/New Caledonia/20/99 (H1N1)) and an H5N1 strain at MOI = 0.1 and cultured at 37 ^o^C showed similar growth rates, with the H5N1 strain peaking earlier at approximately 24 h p.i. while the H1N1 strain peaked later at approximately 70 h p.i^29^. In a study comparing the growth properties of H5N1 of 3 different clades (2.3.2, 2.3.4 and 7), Sun *et al.*^30^ found significant differences in replication between H5N1 strains of different clades with the clade 2.3.4 replicating faster and to higher titer than the other viruses. The strain used in our study (A/Indonesia/5/2005 H5N1) belongs to Clade 2.1^58^. Expanding our modeling approach to H5N1 viruses of different clades could be useful to uncover differences in replication strategies for viruses of the same subtypes but with different potentials for human infections. Overall, our results are in line with the current literature as we observed peak titers in the 36–48h range when infecting A549 cells at a low MOI. The disparity in methods used to assess *in vitro* replication capacities highlighted in the above examples (i.e. different MOIs, cell lines, culture temperature, etc) is a good argument in favour of adding infection modeling to more traditional characterization experiments. A future avenue for our work will also be to implement our modeling approach to better understand the mechanistic differences driving the differential replication of human and avian IAV at those temperatures.

By using a simple mathematical model of *in vitro* infection, we have been able to highlight key differences in the replication parameters of mild seasonal and pandemic H1N1 strains compared to H5N1 and H7N9 virus strains that cause significant morbidity and mortality in humans. The pH1N1 virus had a significantly longer eclipse phase compared to the sH1N1, H5N1 and H7N9 viruses. This is consistent with the independent finding that its non-structural 1 (NS1) protein was detected last (at 6h post-infection instead of 3h) compared to the three other strains^59^. As this protein is not carried by the virions and is expressed early during infection^60^,^61^ it is useful as a marker of *de novo* viral replication. In earlier work, we also found that the length of the eclipse phase was a good indicator of the efficiency of virus release and/or the efficiency of the neuraminidase activity of the strain^44^,^45^. Our results could indicate that, in A549 infections, the virion progeny of the sH1N1, H5N1 and H7N9 viruses are released more easily from the producing cell than that of the pH1N1 virus, potentially pointing to higher neuraminidase and/or lower hemagglutinin activities.

**Figure 3:**
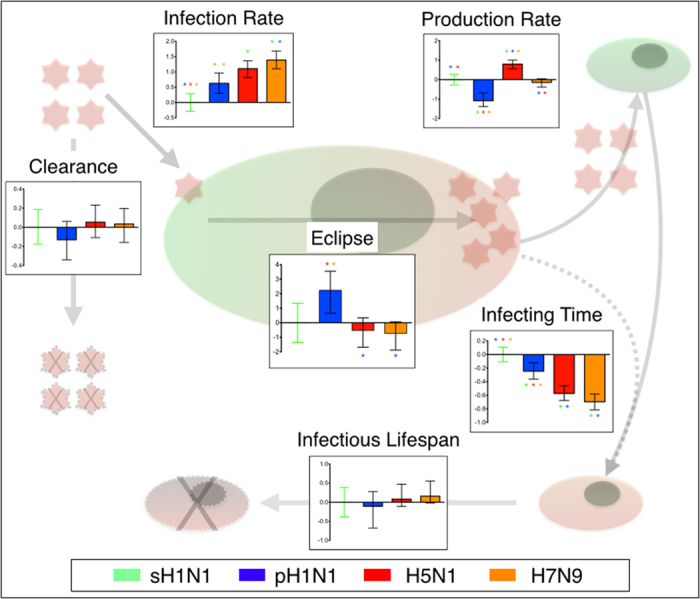
Summary of replication parameters. A simplified representation of the parameters as defined by the model is shown as a ratio of each parameter to the sH1N1 strain. Color coding of bar graph as follows: Reference: Green – sH1N1; Blue – pH1N1; Red – H5N1; Orange – H7N9. Asterisks indicate statistically-significant values (see Table 1). Green cells are non-infected, red cells are infected. X indicates a dead cell or a decayed virion. The computation of these ratios is described in the Methods section.

The two viruses highly pathogenic in humans, the H5N1 and H7N9 viruses, have a marginally shorter eclipse phase and generate a significantly larger number of infections per hour, the two indicators of the overall capacity of a virus to cause rapid infection. As such, the H5N1 and H7N9 viruses had an enhanced capacity for infection of A549 cells compared to the H1N1 viruses. What was also striking is that the larger number of infections per hour caused by the H5N1 and H7N9 viruses can be attributed to a different mechanism for each virus. The H5N1 virus exhibits the highest virus production rate, that is, it produces infectious progeny significantly faster than the three other viruses. In contrast, the H7N9 virus produces infectious progeny only marginally faster than the H1N1 strains, but its progeny is remarkably effective, infecting cells significantly faster per infectious virus unit (per TCID_50_) than the three other viruses. Figure 3 summarizes the differences for each parameter for the four strains in the context of viral replication. The H7N9 virus strain used in this study, Anhui/1/13, has a mixed affinity for α(2,3) and α(2,6)-linked sialic acid (SA)^27^ while the H5N1 virus strain used, Indonesia/5/2005, has a more restrictive affinity for the α(2,3) SA species. The seasonal and pandemic H1N1 strains (sH1N1 and pH1N1) both have the typical α(2.6) tropism found in IAV capable of sustained human-to-human transmission. A549 cells have been shown to express both α(2,3) and α(2,6)-linked sialic acids on their surface glycoproteins^62^. Having a cell line expressing both SA species is useful in our modelling approach as it means that all strains have equal opportunities to attach and infect the cell irrespective of their SA preferences. Therefore, the source of the disparity between the H5N1 and H7N9 viruses infection capacity could be more complex than affinity based on differential receptor binding properties. To isolate whether attachment of the viruses to SA moieties plays a crucial role in explaining the difference in growth parameters, one could design an experiment to specifically block α(2,3) or α(2,6) SA on A549 cells and model the infection output with strains of different tropism. Another approach would be to generate cell lines specifically expressing only one of the SA species and a reporter gene marker (e.g. GFP, mCherry), co-culturing them and using FACS to assess the capacity of various strains to infect both types in single and multi-cycle experiments. Approached as a kinetic experiment and coupled with our model, this could allow the determination of infection rates as a function of the SA species present on the cells. Interestingly, a proteomic study measuring the host response of the same four virus strains in A549 cells showed that overall, the H5N1 and H7N9 strains induced the most profound changes to the cellular proteome and affected the greatest number of metabolic pathways^59^. This could be the cause or an effect of its significantly higher rate of infectious virus production.

The available therapeutic agents for treating influenza infections were developed by identifying key viral proteins and their essential functions in viral replication. Neuraminidase inhibitors^63^,^64^ and M2 ion channel inhibitors^65^,^66^ are the only approved therapeutic options although widespread resistance and significant side-effects are reported with the latter^67^. Current efforts to understand – and disrupt - the differential ability of influenza viruses to cause disease have increasingly shifted to approaches that integrate virology and systems biology. Understanding the differences in how strains of varying pathogenicity undergo each step of the replication cycle could be useful in targeting those specific replication steps which give the most advantage to the virus. The experimentally observed growth kinetics alone (see Fig. 1) clearly demonstrate that the H5N1 and H7N9 viruses have a replicative advantage over the H1N1 strains. But additional analysis with a mathematical model dissected these observations further into detailed, quantitative measures of the replication parameters underlying the processes which give rise to the observed kinetics. The extraction of these parameters using classical virological methods for growth analyses alone would be impossible, or otherwise difficult and remarkably costly. By combining experimental virology assays with mathematical modeling, we have described differences in growth properties from analysis in an *in vitro* system between viruses that exhibit considerable differences in their virulence in human infections. Furthermore, we have demonstrated these viruses, even those that show similar virulence, may differ considerably in their replicative strategies. While it is an interesting first step to delineate the replicative advantages shown by the H5N1 and H7N9 viruses that exhibit great pathogenicity in human infections, further study will be essential to provide understanding of the significance of these distinctions between viruses and their ability to cause a range of disease manifestations.

## Methods

### Virus and cells

Four IAV strains were used in this study. Seasonal H1N1 strain A/Canada/RV733/2003 (sH1N1, A/New Caledonia/20/1999-like clinical isolate) and 2009 H1N1 pandemic strain A/Mexico/INDRE4487/2009 (pH1N1) were chosen to represent low pathogenic, human IAV. Avian influenza strain A/Indonesia/05/2005 (H5N1) represents a highly virulent HPAI strain, while influenza A/Anhui/1/2013 (H7N9) is an early human isolate LPAI from the current Chinese epizootic of an avian IAV. The pH1N1 virus was isolated by our group at the Public Health Agency of Canada (PHAC) and the H5N1 strain was previously generated by us by reverse genetics. The sH1N1 and H7N9 strains were a kind gift of the Respiratory Virus group at PHAC. Stocks of viruses were grown in Madin Darby canine kidney (MDCK – obtained from the American Type Culture Collection, ATCC) cells in MEM containing 0.1% BSA and 1 μg/mL TPCK-treated trypsin. Supernatants where harvested from 10 T150 flasks, concentrated by ultracentrifugation and titrated using standard plaque forming unit (PFU) method (MDCK) cells^68^. All work with H5N1 and H7N9 IAVs was done in a biosafety level 3 laboratory at the National Microbiology Laboratory following Public Health Agency of Canada procedures and regulations.

### Infections

Infections (MC and SC assays) were carried out in A549 human lung carcinoma cells (ATCC) seeded in T25 flasks in triplicate. Stock cells were grown in T150 flasks (Corning) in F12K media (HyClone) supplemented with 5% FBS (Sigma). Upon reaching 90% confluence, cells where harvested and counted in an automatic Countess cell counter (Invitrogen). One million cells where seeded in T25 flasks (Corning) and incubated for 48h. On the days of infection, cells were washed with serum-free F12K media, and virus dilutions (prepared in F12K supplemented with 0.1% BSA and 0.5 μg/mL of TPCK-treated trypsin) were adsorbed onto the 90–95% confluent cells for 1h. All cultures were maintained in a humidified incubator at 37 ^o^C and 5% CO_2_ to maintain the optimal physiological pH for the cells in the range of 7.0–7.4. For each virus in each assay, three separate flasks were infected and prior to all infections 2 spare flasks were trypsinized and counted to ensure accurate MOI. At set time points, 0.5 mL of the 10 mL supernatant volume was harvested, frozen for later quantification, and replaced with 0.5 mL of fresh media (F12K + 0.1% BSA + 0.5 μg/mL TPCK-treated trypsin). Subsequently, the frozen samples were thawed and their virus concentration was quantified via a TCID_50_ titration assay, or for total viral RNA content via quantitative reverse-transcription PCR (qRT-PCR). We used multiplicities of infection (MOI) of 0.01 PFU/cell for the multiple-cycle (MC) infection assay, and an MOI of 3 PFU/cell for the single-cycle (SC) infection assay. For the SC, we used an acidic saline wash^44^. Briefly after adsorption virus inoculum was removed and 1 mL of a warm 0.9% NaCl in cell-culture grade water at pH 2.2 was added. Flasks were rinsed for 10–15 seconds and this was removed and washed 4 times with F12K media. A mock-yield (MY) assay was also performed where virus was left to decay in cell-free media. In this assay, 10^7^ PFU of each strains was added to 7 mL of F12K media and 0.1% BSA in three separate T25 flasks without any cells. This was incubated at 37 ^o^C in a 5% CO_2_ atmosphere and aliquots were harvested for titration every 24 h for four days.

### qRT-PCR

RNA extractions were done following the Qiagen ViralRNA spin protocol. For qRT-PCR, the one-step Roche Probe Hydrolysis kit with a LightCycler 480 instrument and the LightCycler software (version SW 1.5.1) was used with a universal primer-probe set able to quantify the M gene of all IAVs (Forward primer: 5-GACCRATCCTGTCACCTCTGAC-3, Reverse primer: 5-AGGGCATTYTGCACAAAKCGTCTA-3, Probe: 5-FAM-TGCAGTCCTCGCTCACTGGGCACG-BHQ1-3). As reported elsewhere^69^, this set is not optimal for the pH1N1 strain, and so primer sets to quantify the HA and NA of the pH1N1 strains were also used (Forward HA primer: 5-TGGCTGGATCCTGGGAAATC-3; Reverse HA primer: 5-CGATGAAATCTCCTGGGTAACAC-3; HA Probe: 5-FAM-CACTCTCCACAGCAAGCTCATGGTCCTAC-BHQ1-3) (Forward NA primer: 5-TTAACATCAGCAACACCAACTTTG-3; Reverse NA primer: 5- CCATCCACTAACAGGGCAGAG-3; NA Probe: 5-FAM-CACTCTCCACAGCAAGCTCATGGTCCTAC-BHQ1-3). For the pH1N1 strain, qRT-PCR was performed with all three primer sets (M, HA and NA) for the SC assay. The M primers were used for the MC assay, and the MC copy numbers were converted to that obtained with HA and NA primers based on the HA:M and NA:M ratios determined in the SC assay. Primers and probes were purchased from IDT and the pH1N1 HA and NA primer/probe sets were kindly designed and donated by Dr. Yan Li at the Public Health Agency of Canada. The standard curves for measuring copy numbers were determined using plasmids containing the whole M, HA or NA gene of each respective viruses (i.e. sH1N1-M, pH1N1-M, H5N1-M, H7N9-M, pH1N1-HA and pH1N1-NA).

### Mathematical modelling

The SC and MC *in vitro* infection experiments were simulated numerically using an age-structured, ordinary differential equation (ODE) mathematical model, introduced and described previously^45^,^50^. The ODE model, Eqn. (1), considers both the infectious (*V*_TCID50_) and total (*V*_RNA_) virus concentration released by infectious cells into the supernatant over the course of the *in vitro* infections.


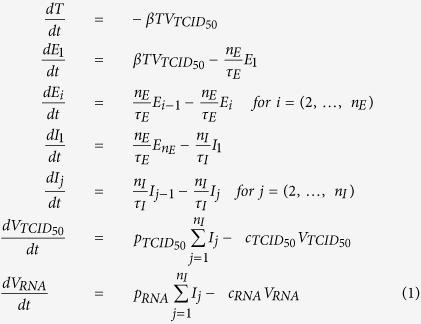


The mathematical model represents cells in the uninfected, target state (*T*) becoming infected by infectious virus (V_TCID50_) at rate βV_TCID50_. The newly infected cells first undergo an eclipse phase (*E*_*i*_) of duration *τ*_*E*_(1 ± 1/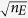) during which time they cannot yet produce and release virus. In time, these infected cells leave the eclipse phase to enter the infectious phase (*I*_*j*_) during which time they produce total (V_RNA_) and infectious (V_TCID50_) virus at constant rates *p*_RNA_ and 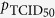, respectively, for a time *τ*_*I*_(1 ± 1/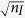), until they cease virus production and possibly undergo apoptosis. The infectious virus (V_TCID50_) progeny is assumed to lose infectivity (so as to be undetectable via tissue culture) at exponential rate 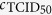, whilst the total virus (V_RNA_) progeny is assumed to degrade (so as to be unmeasurable by qRT-PCR) at rate *c*_*RNA*_.

In the mathematical model (Eqn. 1), an Erlang distribution is used to represent the time spent by cells in the eclipse and infectious phases by using a series of ODEs or age-classes. The number of ODEs or age-classes (*n*_*E*_ and *n*_*I*_) — which is equal to the Erlang distribution’s shape parameter — can be used to select whether the time duration of the phase will be distributed according to an exponential (1 age-class), a normal (≫ one age-class), or even a Dirac delta (as the number of age-classes tends to infinity)^70^,^71^,^72^. We have shown in previous work that exponential (*n*_*E*_ or *n*_*I*_ = 1) and Dirac delta (very large *n*_*E*_ or *n*_*I*_) distributions for the eclipse or infectious phases cannot reproduce the viral yield kinetics of the single-cycle experiment for influenza, and are therefore inappropriate in the present context^73^,^50^. As for using an intermediate number of compartments (1 < [*n*_*E*_ or *n*_*I*_] ≪ 1000), i.e. slowly shifting from an exponential-like to a normal-like distribution, we have explained in previous work^50^ that in doing so the goodness-of-fit (see below for a definition of the goodness-of-fit) improves as *n*_*E*_ or *n*_*I*_ are increased, up until approximately > 40–50 equations, past which point the model is largely insensitive to the specific values of *n*_*E*_ or *n*_*I*_ used. For this reason, here and in previous work, we have chosen to fix *n*_*E*_ = *n*_*I*_ = 60^50^.

The application of this mathematical model to reproduce the course of SC and MC infections and MY assay, and to extract the key infection parameters characterizing the replication fitness of an IAV strain, has been described in detail elsewhere^45^,^50^, but specific details are repeated here for clarity and completeness. Eqn. (1) was used to simulate the SC and MC experiments, using the same initial conditions for cell, namely by initiating infection with 100% of cells susceptible to infection, *T*(*t*_*start*_) = 1, and no initially infected cells, *E*_*i*_(*t*_start_) = *I*_*j*_(*t*_start_) = 0. In simulating the MC experiment, the initial conditions for the total (V_RNA_) and infectious (V_TCID50_) virus inoculum concentration at time *t*_start_ = 0 h were fixed to the geometric mean of the experimentally measured concentrations at time points up to 7 h post-infection (hpi) for each strain, and the inoculum was not rinsed in the MC experiment. In simulating the SC experiment, the initial condition for the infectious virus (V_TCID50_) at time *t*_start_ =  −1 h was computed using


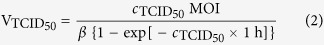


where MOI = 3 [infectious virion/cell] is the multiplicity of infection in the SC inoculum. The total virus concentration (V_RNA_) was set to an arbitrary value at *t*_start_ =  −1 h as it does not contribute to infection; it is only tracked in the model for the purpose of comparing it against experimental measurements and it was not measured during the incubation period (t = −1 h to 0 h). After a 1h incubation period (i.e. at time t = 0), the experimental rinsing of the viral inoculum was simulated in the mathematical model by setting the total (V_RNA_) and infectious (V_TCID50_) virus concentration in the model to the geometric mean of the experimentally measured concentrations at time points up to t = 3.5 h p.i. for each strain.

In addition to the initial conditions outlined above, the mathematical model in Eqn. (1) has 9 parameters (*β*, *p*_RNA_, 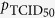, *c*_RNA_, 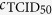, *τ*_*E*_, *n*_*E*_, *τ*_*I*_, *n*_*I*_), and for a given strain, the SC and MC experiments were simulated using the same value for these 9 parameters, with the exception of the rate of infectious virus production, 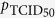. Parameter 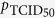 was allowed to differ between the SC and MC experiment, as in our previous work^50^, to account for potential viral yield reduction (a smaller rate of infectious virus production, 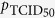) in the SC experiment which would arise when using an inoculum of high MOI if defective interfering particles (DIPs) were present in our samples. Additionally, the rate of viral RNA degradation was fixed at *c*_RNA_ = 0.001/h, and the number of eclipse and infectious compartments were fixed to *n*_*E*_ = *n*_*I*_ = 60, as established in previous work^50^. This left a total of 7 parameters (*β*, *p*_RNA_, 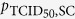, 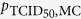, 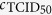, *τ*_*E*_, *τ*_*I*_) whose values were to be determined from the SC, MC, and MY experimental data as described in the next section.

### Determination of model parameter distributions from the experimental data

The goodness-of-fit between the mathematical model in Eqn. (1) and the combined experimental data for the SC, MC and MY experiments was computed via a sum of squared residuals (SSR) as follows:


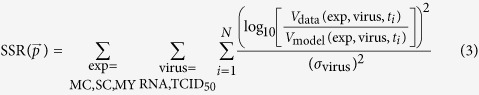


where SSR(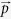) is the sum-of-squared residuals between the combined experimental data and the model’s predictions given a particular set of parameter values (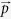), *σ*_RNA_ = 1 RNA/mL, 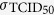 = 1 TCID_50_/mL, and the virus concentration V_model_(MY, virus) was computed as described previously^50^, using


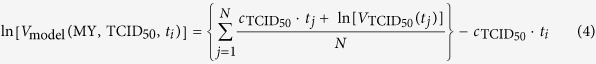


For each of the four strains, a nonlinear regression was initially performed to find a parameter set which minimized the SSR (Eqn. 3) by sequentially using Python scipy’s Nelder-Mead (scipy.optimize.fmin) and Levenberg-Marquardt (scipy.optimize.leasqr) implementations to find the parameters, and function scipy.integrate.odeint to numerically solve Eqn. (1).

Next, a Markov Chain Monte Carlo simulation with 300 walkers (or chains) performing 2000 steps was executed using the python emcee module^74^. The starting position of the 300 walkers (i.e. the parameters’ prior distributions) for each strain was chosen from a logarithmically uniform distribution, centred on the parameter values determined via the abovementioned nonlinear regression (hereafter *f*_base_), with a width of one order of magnitude, namely *f*_base_ ⋅ 10^[−1,1]^, for all parameters except τ_*E*_. Because past work has shown that τ_*E*_, the length of the eclipse phase, is typically normally distributed rather than log-normally distributed^50^, a linearly uniform distribution, namely *τ*_*E*_ ∈ *f*_*base*_ ⋅ [1,3]/2, was used instead. The likelihood (probability function) of accepting a particular step (parameter set value proposition) was defined as exp[−SSR(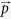)]. Of the 2000 steps performed for each strain, 250 steps were discarded as burn-in, resulting in a total of 525,000 (1750 steps ∙ 300 walkers) accepted parameter sets which formed our posterior parameter distributions.

Analysis of the posterior distributions for the model parameters revealed a number of correlations, and more parameter correlations were found for the two H1N1 viruses than for the two avian viruses (H5N1, H7N9), as shown in Supplementary Fig. 1. Across all four viruses studied, there was a clear correlation between the infectious virus production rate (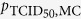) and the virus effectiveness (β), due to the equivalence of having more virus (higher production rate) that is less infectious (lower effectiveness), or having less virus that is more infectious, in maintaining the same rate of disease progression, i.e. new infections caused per hour. This correlation and others like it mean that when the experimental data only weakly constrains one of the two parameters (e.g. due to experimental variability), the variability of both parameters will be increased, resulting in wider posterior distributions for these parameters. A wider distribution translates to a reduced sensitivity in our ability to detect statistically significant differences in that parameter between the 4 strains studied. In other words, if the correlations could be broken or avoided somehow with additional data or complementary experiments, the two-dimensional histograms in Supplementary Fig. 1 would become symmetric and more constrained, leading to narrower posterior distributions, and a greater number of parameter differences between strains would become statistically significant (*p*-value < 0.05). The near absence of correlations between the parameters extracted for the two avian viruses suggests that the set of experiments conducted provided an appropriate set of complementary information which, when experimental variability is reasonably small, can appropriately constrain model parameters and eliminate correlations. Unfortunately, the greater experimental variability in the H1N1 strains weakened these constraints and likely caused the larger number of correlations and the wider posterior distributions for certain parameters. In particular, the length of the eclipse phase (*τ*_*E*_) showed a positive correlation to the viral production rate (*p*_*RNA*_). This in turn limited the precision with which we could determine these parameters: the uncertainty on the eclipse phase is much larger for the H1N1 strains than the H5N1 and H7N9 strains. This could be because the two H1N1 virus infections progressed more slowly, involved fewer virions, such that the infection kinetics could be more susceptible to small, stochastic effects, resulting in larger experimental variability. In contrast, the H5N1 and H7N9 strains are characterized by larger production rates. This more rapidly separates the signal from the inherent noise within the data, and allows for a more precise determination of the eclipse phase (*τ*_*E*_). With regards to the H1N1 strains, novel laboratory techniques would be required to reduce the data variability so as to eliminate such correlations between parameters. Nonetheless, the parameter differences we highlight herein were statistically significant, and when they were not, we either state that the change is marginal or do not mention it. None of the statistically significant differences we highlight between pairs of parameters would cease to be so in the absence of correlations. The parameter differences we highlight are statistically significant in the presence of, and despite, sometimes wide parameter posterior distributions which were occasionally the result of parameter correlations. In the absence of correlations, these differences would only have become more significant, and some differences that are currently not so, would become so.

In Table 1, statistically significant differences in the values of each parameter between pairs of strains were reported as *p*-values, computed as per the following example. Say the median value of parameter *f* is larger for virus A than for virus B. Then the *p*-value for (the statistical likelihood of) parameter *f* being larger for virus A than virus B was computed in a non-parametric manner by drawing, at random and with replacement, one parameter value from virus A’s and one from virus B’s 525,000 accepted values for parameter *f*, and determining what fraction of the draws in which the value of *f* drawn from strain A was greater than that from strain B after 2,625,000 such random draws. For normally distributed posterior parameter distributions, the *p*-value obtained by this method is equal to that obtained using a one-tailed, Student t-test.

In Fig. 3, the ratios of the parameter values for the pH1N1, H5N1, and H7N9 strains to that of the sH1N1 strains were computed as per the following example. The ratio of parameter *f* for virus B to that of the sH1N1 virus was determined by drawing, at random and with replacement, one parameter value from virus B’s and one from the sH1N1 virus’ 525,000 accepted values for parameter *f*, calculating their ratio (*f*_B_/*f*_sH1N1_), and repeating this procedure for 525,000 such random draws. Figure 3 shows the non-parametric median (the coloured bars) and the one-sigma (68.3% credible region) variability (error bars) of the resulting set of (*f*_B_/*f*_sH1N1_) ratios for a given parameter *f*.

## Additional Information

**How to cite this article**: Simon, P. F. *et al.* Avian influenza viruses that cause highly virulent infections in humans exhibit distinct replicative properties in contrast to human H1N1 viruses. *Sci. Rep.* **6**, 24154; doi: 10.1038/srep24154 (2016).

## Supplementary Material

Supplementary Information

## Figures and Tables

**Table 1:** Parameters characterizing the replication efficacy of each strain.

Parameter Values [95% credible region]			sH1N1	pH1N1	H5N1	H7N9
Degradation rate, 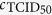 (1/h)			0.0573 [0.03, 0.098]	0.0414 [0.017, 0.079]	0.0657 [0.038, 0.1]	0.0596 [0.032, 0.098]
Eclipse phase duration, *τ*_*E*_ (h)			7.04 [5.6, 9.7]	9.15 [6.9, 11]	6.27 [5.5, 7.1]	6.05 [5.4, 6.7]
Prod. Infectious virus, 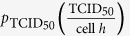			10^1.11 [0.67, 1.8]^	10^1.21 [0.63, 2]^	10^2.06 [1.7, 2.4]^	10^1.41 [1, 1.8]^
Virus effectiveness, 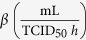			10^−6.3 [−6.8, −5.9]^	10^−5.94 [−6.5, −5.3]^	10^−6.1 [−6.5, −5.7]^	10^−5.19 [−5.6, −4.7]^
Rate of infection, 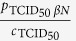 (1/h)			10^1.34 [1, 1.8]^	10^1.97 [1.5, 2.6]^	10^2.42 [2.1, 2.8]^	10^2.72 [2.3, 3.1]^
Pairwise statistical significance (*p*-values)						
	sH1N1:pH1N1	sH1N1:H5N1	sH1N1:H7N9	pH1N1:H5N1	pH1N1:H7N9	H5N1:H7N9
Degradation rate (1/h)	0.233	0.364	0.466	0.140	0.209	0.396
Eclipse phase duration (h)	0.105	0.201	0.122	**0.010**	**0.004**	0.339
Prod. infectious virus 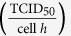	0.418	**0.007**	0.201	**0.033**	0.332	**0.007**
Virus effectiveness 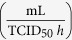	0.171	0.259	**<0.001**	0.339	**0.027**	**0.002**
Rate of infection (1/h)	**0.033**	**<0.001**	**<0.001**	0.088	**0.018**	0.135

The mode and 95% credible region (analogous to the 95% confidence interval) for each parameter distribution are shown. The bottom table indicates differences of statistical significance (*p*-values) between each pair of viruses, computed as described in the Methods section. The virological meaning of each parameter is explained in Fig. 2.

## References

[b1] Wright P. M., Neumann G. & Kawaoka Y. In Fields Virology 6th edn, Vol. 1 (eds. Knipe D. M. & Howley P. M. ) Ch. 41, 1186–1243 (Lippincott Williams & Wilkins, 2013).

[b2] Shaw M. L. & Palese P. In Fields Virology 6th edn Vol. 1 (eds. Knipe D. M. & Howley P. M. ) Ch. 40, 1151–1185 (Lippincott Williams & Wilkins, 2013).

[b3] Nicholson K. G., Wood J. M. & Zambon M. Influenza. Lancet 362, 1733–1745 (2003). 14643124 10.1016/S0140-6736(03)14854-4 PMC7112395

[b4] Cox N. J. & Subbarao K. Influenza. Lancet 354, 1277–1282 (1999). 10520648 10.1016/S0140-6736(99)01241-6

[b5] Morens D. M., Taubenberger J. K. & Fauci A. S. The persistent legacy of the 1918 influenza virus. N. Engl. J. Med. 361, 225–9 (2009). 19564629 10.1056/NEJMp0904819 PMC2749954

[b6] Broor S. *et al.* Dynamic patterns of circulating seasonal and pandemic A(H1N1)pdm09 influenza viruses from 2007–2010 in and around Delhi, India. PLoS One 7, e29129 (2012). 22235265 10.1371/journal.pone.0029129 PMC3250412

[b7] Blyth C. C. *et al.* The impact of the pandemic influenza A(H1N1) 2009 virus on seasonal influenza A viruses in the southern hemisphere, 2009. Eurosurveillance 15, 1–5 (2010). 20738990

[b8] Poovorawan Y., Pyungporn S., Prachayangprecha S. & Makkoch J. Global alert to avian influenza virus infection: from H5N1 to H7N9. Pathog. Glob. Health 107, 217–23 (2013). 23916331 10.1179/2047773213Y.0000000103 PMC4001451

[b9] Yoon S.-W., Webby R. J. & Webster R. G. Evolution and ecology of influenza A viruses. Curr. Top. Microbiol. Immunol. 385, 359–75 (2014). 24990620 10.1007/82_2014_396

[b10] Wu Y., Wu Y., Tefsen B., Shi Y. & Gao G. F. Bat-derived influenza-like viruses H17N10 and H18N11. Trends Microbiol. 22, 183–191 (2014). 24582528 10.1016/j.tim.2014.01.010 PMC7127364

[b11] Center for Disease Control and Prevention. Highly Pathogenic Avian Influenza A (H5N1) in People | Avian Influenza (Flu). http://www.cdc.gov/flu/avianflu/h5n1-people.htm, (2014) (accessed 20/01/2016).

[b12] Center for Disease Control and Prevention. Isolation of avian influenza A(H5N1) viruses from humans–Hong Kong, May-December 1997. Morb. Mortal. Wkly. Rep. **46**, 1204–1207 (1997). 9414153

[b13] Payton L. H5N1 bird flu death confirmed in Alberta, 1st in North America - Politics - CBC News. http://www.cbc.ca/news/politics/h5n1-bird-flu-death-confirmed-in-alberta-1st-in-north-america-1.2489160, (2014) (20/01/2016).

[b14] FluTrackers. China - Hong Kong closely monitors 4 additional human cases of H7N9 in Mainland - 2 new cases in Guangdong - January 23, 2015. https://flutrackers.com/forum/forum/china-h7n9-outbreak-tracking/h7n9-who-wpro-ecdc-oie-fao-moa-chp-cnfpc-reports-and-updates/722181-china-hong-kong-closely-monitors-4-additional-human-cases-of-h7n9-in-mainland-2-new-cases-in-guangdong-january-23-2015, (2015) (accessed 20/01/2016).

[b15] World Health Organization. Human infection with influenza A(H7N9) virus in China - 1 April 2013. *Global Alert and Response (GAR)*. http://www.who.int/csr/don/2013_04_01/en/, (2013) (accessed 20/01/2016).

[b16] World Health Organization. Human infection with avian influenza A(H7N9) virus – China. *Emergencies preparedness, response*. http://www.who.int/csr/don/11-march-2015-avian-influenza-china/en/, (2015) (accessed 20/01/2016).

[b17] World Health Organization. WHO | Human infection with avian influenza A(H7N9) virus – Canada. *Emergencies preparedness, response*. http://www.who.int/csr/don/01-february-2015-avian-influenza/en/, (2015) (accessed 20/01/2016).

[b18] Morens D. M., Taubenberger J. K. & Fauci A. S. H7N9 avian influenza A virus and the perpetual challenge of potential human pandemicity. MBio 4, 3–6 (2013). 10.1128/mBio.00445-13 PMC3705455 23839219

[b19] Böttcher-Friebertshäuser E., Garten W., Matrosovich M. & Klenk H. D. The hemagglutinin: a determinant of pathogenicity. Curr. Top. Microbiol. Immunol. 351, 3–34 (2014). 10.1007/82_2014_384 25031010

[b20] Kobasa D. *et al.* Enhanced virulence of influenza A viruses with the haemagglutinin of the 1918 pandemic virus. Nature 431, 703–707 (2004). 15470432 10.1038/nature02951

[b21] Herfst S. *et al.* Airborne transmission of influenza A/H5N1 virus between ferrets. Science 336, 1534–41 (2012). 22723413 10.1126/science.1213362 PMC4810786

[b22] Russell C. a *et al.* The potential for respiratory droplet-transmissible A/H5N1 influenza virus to evolve in a mammalian host. Science 336, 1541–7 (2012). 22723414 10.1126/science.1222526 PMC3426314

[b23] Simon P. *et al.* The I222V neuraminidase mutation has a compensatory role in replication of an oseltamivir-resistant influenza virus A/H3N2 E119V mutant. J. Clin. Microbiol. 49, 715–7 (2011). 21106781 10.1128/JCM.01732-10 PMC3043469

[b24] Baz M., Abed Y., Simon P., Hamelin M.-E. & Boivin G. Effect of the neuraminidase mutation H274Y conferring resistance to oseltamivir on the replicative capacity and virulence of old and recent human influenza A(H1N1) viruses. J. Infect. Dis. 201, 740–5 (2010). 20100088 10.1086/650464

[b25] Hamelin M.-E. *et al.* Oseltamivir-resistant pandemic A/H1N1 virus is as virulent as its wild-type counterpart in mice and ferrets. PLoS Pathog. 6, e1001015 (2010). 20661429 10.1371/journal.ppat.1001015 PMC2908621

[b26] Itoh Y. *et al.* *In vitro* and *in vivo* characterization of new swine-origin H1N1 influenza viruses. Nature 460, 1021–1025 (2009). 19672242 10.1038/nature08260 PMC2748827

[b27] Watanabe T. *et al.* Characterization of H7N9 influenza A viruses isolated from humans. Nature 501, 551–5 (2013). 23842494 10.1038/nature12392 PMC3891892

[b28] Zhang X. *et al.* Hemagglutinin glycosylation modulates the pathogenicity and antigenicity of the H5N1 avian influenza virus. Vet. Microbiol. 175, 244–56 (2015). 25544041 10.1016/j.vetmic.2014.12.011

[b29] Josset L. *et al.* Gene expression signature-based screening identifies new broadly effective influenza a antivirals. PLoS One 5, 18 (2010). 10.1371/journal.pone.0013169 PMC2949399 20957181

[b30] Sun H. *et al.* Comparative virus replication and host innate responses in human cells infected with three prevalent clades (2.3.4, 2.3.2, and 7) of highly pathogenic avian influenza H5N1 viruses. J. Virol. 88, 725–9 (2014). 24131718 10.1128/JVI.02510-13 PMC3911739

[b31] Yamaji R. *et al.* Identification of PB2 Mutations Responsible for the Efficient Replication of H5N1 Influenza Viruses in Human Lung Epithelial Cells. J. Virol. 89, 3947–3956 (2015). 25609813 10.1128/JVI.03328-14 PMC4403392

[b32] Mitchell H. *et al.* Higher level of replication efficiency of 2009 (H1N1) pandemic influenza virus than those of seasonal and avian strains: kinetics from epithelial cell culture and computational modeling. J. Virol. 85, 1125–35 (2011). 21068247 10.1128/JVI.01722-10 PMC3019989

[b33] Hoopes J. D. *et al.* Triple combination antiviral drug (TCAD) composed of amantadine, oseltamivir, and ribavirin impedes the selection of drug-resistant influenza A virus. PLoS One 6, e29778 (2011). 22220216 10.1371/journal.pone.0029778 PMC3248427

[b34] Hur K.-Y., Moon J.-Y., Kim S.-H. & Yoo J.-Y. Model-based simulation and prediction of an antiviral strategy against influenza A infection. PLoS One 8, e68235 (2013). 23874556 10.1371/journal.pone.0068235 PMC3706530

[b35] Petrie S. M. *et al.* Reducing Uncertainty in Within-Host Parameter Estimates of Influenza Infection by Measuring Both Infectious and Total Viral Load. PLoS One 8, e64098 (2013). 23691157 10.1371/journal.pone.0064098 PMC3655064

[b36] Howat T. J., Barreca C., O’Hare P., Gog J. R. & Grenfell B. T. Modelling dynamics of the type I interferon response to *in vitro* viral infection. J. R. Soc. Interface 3, 699–709 (2006). 16971338 10.1098/rsif.2006.0136 PMC1664656

[b37] Holder B. P., Liao L. E., Simon P., Boivin G. & Beauchemin C. A. A. Design considerations in building in silico equivalents of common experimental influenza virus assays. Autoimmunity 44, 282–293 (2011). 21244331 10.3109/08916934.2011.523267

[b38] Capasso V. & Maddalena L. Convergence to equilibrium states for a reaction-diffusion system modelling the spatial spread of a class of bacterial and viral diseases. J. Math. Biol. 13, 173–84 (1981). 7328365 10.1007/BF00275212

[b39] Perelson A. S. Modelling viral and immune system dynamics. Nat. Rev. Immunol. 2, 28–36 (2002). 11905835 10.1038/nri700

[b40] Borghans J. a, De Boer R. J., Sercarz E. & Kumar V. T cell vaccination in experimental autoimmune encephalomyelitis: a mathematical model. J. Immunol. 161, 1087–93 (1998). 9686566

[b41] Perelson A. S., Neumann A. U., Markowitz M., Leonard J. M. & Ho D. D. HIV-1 dynamics *in vivo*: virion clearance rate, infected cell life-span, and viral generation time. Science 271, 1582–1586 (1996). 8599114 10.1126/science.271.5255.1582

[b42] Neumann A. U. *et al.* Hepatitis C viral dynamics *in vivo* and the antiviral efficacy of interferon-therapy. Science 282, 103 (1998). 9756471 10.1126/science.282.5386.103

[b43] Nowak M. & May R. M. Virus dynamics: mathematical principles of Immunology and virology: mathematical principles of Immunology and virology. pp.1–250 (Oxford university press, 2000).

[b44] Holder B. P. *et al.* Assessing the *In Vitro* Fitness of an Oseltamivir-Resistant Seasonal A/H1N1 Influenza Strain Using a Mathematical Model. PLoS One 6, e14767 (2011). 21455300 10.1371/journal.pone.0014767 PMC3063785

[b45] Pinilla L. T., Holder B. P., Abed Y., Boivin G. & Beauchemin C. A. A. The H275Y Neuraminidase Mutation of the Pandemic A/H1N1 Influenza Virus Lengthens the Eclipse Phase and Reduces Viral Output of Infected Cells, Potentially Compromising Fitness in Ferrets. J. Virol 86, 10651–10660 (2012). 22837199 10.1128/JVI.07244-11 PMC3457267

[b46] Beauchemin C. A. A. *et al.* Modeling amantadine treatment of influenza A virus *in vitro*. J. Theor. Biol. 254, 439–451 (2008). 18653201 10.1016/j.jtbi.2008.05.031 PMC2663526

[b47] Moscona A. Global transmission of oseltamivir-resistant influenza. N. Engl. J. Med. 360, 953–956 (2009). 19258250 10.1056/NEJMp0900648

[b48] Lackenby A., Thompson C. I. & Democratis J. The potential impact of neuraminidase inhibitor resistant influenza. Curr. Opin. Infect. Dis. 21, 626–638 (2008). 18978531 10.1097/QCO.0b013e3283199797

[b49] Correia V., de Andrade H. R., Santos L. A., Lackenby A. & Zambon M. Antiviral drug profile of seasonal influenza viruses circulating in Portugal from 2004/2005 to 2008/2009 winter seasons. Antiviral Res. 86, 128–36 (2010). 20083142 10.1016/j.antiviral.2010.01.002

[b50] Paradis E. G. *et al.* Impact of the H275Y and I223V Mutations in the Neuraminidase of the 2009 Pandemic Influenza Virus *In Vitro* and Evaluating Experimental Reproducibility. PLoS One 10, e0126115 (2015). 25992792 10.1371/journal.pone.0126115 PMC4439092

[b51] Broadbent A. J., Santos C. P., Godbout R. A. & Subbarao K. The temperature-sensitive and attenuation phenotypes conferred by mutations in the influenza virus PB2, PB1, and NP genes are influenced by the species of origin of the PB2 gene in reassortant viruses derived from influenza A/California/07/2009 and A/WSN/. J. Virol. 88, 12339–47 (2014). 25122786 10.1128/JVI.02142-14 PMC4248888

[b52] Li I. W. S. *et al.* Differential susceptibility of different cell lines to swine-origin influenza A H1N1, seasonal human influenza A H1N1, and avian influenza A H5N1 viruses. J. Clin. Virol. 46, 325–30 (2009). 19801200 10.1016/j.jcv.2009.09.013

[b53] Zhou B. *et al.* Engineering temperature sensitive live attenuated influenza vaccines from emerging viruses. Vaccine 30, 3691–702 (2012). 22449422 10.1016/j.vaccine.2012.03.025 PMC3595159

[b54] Massin P. *et al.* Temperature sensitivity on growth and/or replication of H1N1, H1N2 and H3N2 influenza A viruses isolated from pigs and birds in mammalian cells. Vet. Microbiol. 142, 232–41 (2010). 19926410 10.1016/j.vetmic.2009.10.012

[b55] Scull M. A. *et al.* Avian Influenza virus glycoproteins restrict virus replication and spread through human airway epithelium at temperatures of the proximal airways. PLoS Pathog. 5, e1000424 (2009). 19436701 10.1371/journal.ppat.1000424 PMC2673688

[b56] Zeng H. *et al.* A(H7N9) virus results in early induction of proinflammatory cytokine responses in both human lung epithelial and endothelial cells and shows increased human adaptation compared with avian H5N1 virus. J. Virol. 89, 4655–67 (2015). 25673714 10.1128/JVI.03095-14 PMC4442366

[b57] Kasloff S. B. & Weingartl H. M. Swine alveolar macrophage cell model allows optimal replication of influenza A viruses regardless of their origin. Virology 490, 91–98 (2016). 26855331 10.1016/j.virol.2016.01.006

[b58] World Health Organization. Antigenic and genetic characteristics of H5N1 viruses and candidate vaccine viruses developed for potential use in human vaccines. 1–6. http://www.who.int/influenza/resources/documents/recommendationvaccine.pdf, (2015) (accessed 20/01/2016).

[b59] Simon P. F. *et al.* Highly Pathogenic H5N1 and Novel H7N9 Influenza A Viruses Induce More Profound Proteomic Host Responses than Seasonal and Pandemic H1N1 Strains. J. Proteome Res. 14, 4511–23 (2015). 10.1021/acs.jproteome.5b00196 26381135

[b60] Rahim M. N. *et al.* Generation and characterization of a new panel of broadly reactive anti-NS1 mAbs for detection of influenza A virus. J. Gen. Virol. 94, 593–605 (2013). 23223621 10.1099/vir.0.046649-0

[b61] Hatada E., Hasegawa M., Mukaigawa J., Shimizu K. & Fukuda R. Control of influenza virus gene expression: quantitative analysis of each viral RNA species in infected cells. J. Biochem. 105, 537–546 (1989). 2760014 10.1093/oxfordjournals.jbchem.a122702

[b62] Kumari K. *et al.* Receptor binding specificity of recent human H3N2 influenza viruses. Virol. J. 4, 42 (2007). 17490484 10.1186/1743-422X-4-42 PMC1876801

[b63] Kim C. U. *et al.* Influenza neuraminidase inhibitors possessing a novel hydrophobic interaction in the enzyme active site: design, synthesis, and structural analysis of carbocyclic sialic acid analogues with potent anti-influenza activity. J. Am. Chem. Soc. 119, 681–690 (1997). 16526129 10.1021/ja963036t

[b64] Von Itzstein M. *et al.* Rational design of potent sialidase-based inhibitors of influenza virus replication. Nature 363, 418–423 (1993). 8502295 10.1038/363418a0

[b65] Davies W. L. *et al.* Antiviral activity of 1-adamantanamine (amantadine). Science 144, 862–3 (1964). 14151624 10.1126/science.144.3620.862

[b66] Bukrinskaya a G., Vorkunova N. K., Kornilayeva G. V, Narmanbetova R. a & Vorkunova G. K. Influenza virus uncoating in infected cells and effect of rimantadine. J. Gen. Virol. 60, 49–59 (1982). 7097250 10.1099/0022-1317-60-1-49

[b67] Fiore A. E. *et al.* Antiviral agents for the treatment and chemoprophylaxis of influenza—recommendations of the Advisory Committee on Immunization Practices (ACIP). MMWR. Recomm. Rep. 60, 1–24 (2011). 21248682

[b68] Matrosovich M., Matrosovich T., Garten W. & Klenk H.-D. New low-viscosity overlay medium for viral plaque assays. Virol. J. 3, 63 (2006). 16945126 10.1186/1743-422X-3-63 PMC1564390

[b69] Chen Y. *et al.* Simultaneous detection of influenza A, influenza B, and respiratory syncytial viruses and subtyping of influenza A H3N2 virus and H1N1 (2009) virus by multiplex real-time PCR. J. Clin. Microbiol. 49, 1653–1656 (2011). 21270233 10.1128/JCM.02184-10 PMC3122825

[b70] Kakizoe Y. *et al.* A method to determine the duration of the eclipse phase for *in vitro* infection with a highly pathogenic SHIV strain. Sci. Rep. 5, 10371 (2015). 25996439 10.1038/srep10371 PMC4440524

[b71] Lloyd A. L. Realistic Distributions of Infectious Periods in Epidemic Models: Changing Patterns of Persistence and Dynamics. Theor. Popul. Biol. 60, 59–71 (2001). 11589638 10.1006/tpbi.2001.1525

[b72] Anderson D. & Watson R. On the Spread of a Disease with Gamma Distributed Latent and Infectious Periods. Biometrika 67, 191 (1980).

[b73] Holder B. P. & Beauchemin C. A. A. Exploring the effect of biological delays in kinetic models of influenza within a host or cell culture. BMC Public Health 11, S10 (2011). 10.1186/1471-2458-11-S1-S10 PMC3317580 21356129

[b74] Foreman-Mackey D., Hogg D. W., Lang D. & Goodman J. emcee: The MCMC hammer. Publ. Astron. Soc. Pacific 125, 306–312 (2013).

