# Supplementary material for: Avian influenza viruses that cause highly virulent infections in humans exhibit distinct replicative properties in contrast to human H1N1 viruses
Source: Sci Rep. 2016 Apr 15;6:24154. doi: 10.1038/srep24154 (PMC4832183; doi:10.1038/srep24154)
Supplement: Supplementary Information [file srep24154-s1.pdf]

Supplementary Materials for:

Avian influenza viruses that cause highly virulent infections in humans exhibit distinct replicative properties in contrast to human H1N1 viruses

by

Philippe F. Simon, Marc-Antoine de La Vega, Éric Paradis, Emelissa Mendoza,
Kevin Coombs, Darwyn Kobasa, and Catherine A. A. Beauchemin

Supplementary Figure S1 (4 pages)

The following 4 pages contain 2-dimensional histograms (contour plots) representing the posterior probability density functions for each of the 21 possible pairs of parameters, given the 7 parameters of the mathematical model (Eqn. 1), for each of the 4 influenza A viruses studied in this work (sH1N1, pH1N1, H5N1, H7N9).

The parameter short-hands used in the following 4 figure panels are as follows:

$$\beta = \text{b}$$

$$p_{\text{RNA}} = \text{pr}$$

$$p_{\text{TCID}_{50},\text{MC}} = \text{pinfmc}$$

$$p_{\text{TCID}_{50},\text{SC}} = \text{pinfsc}$$

$$c_{\text{TCID}_{50}} = \text{cinf}$$

$$\tau_E = \text{tE}$$

$$\tau_I = \text{tI}$$

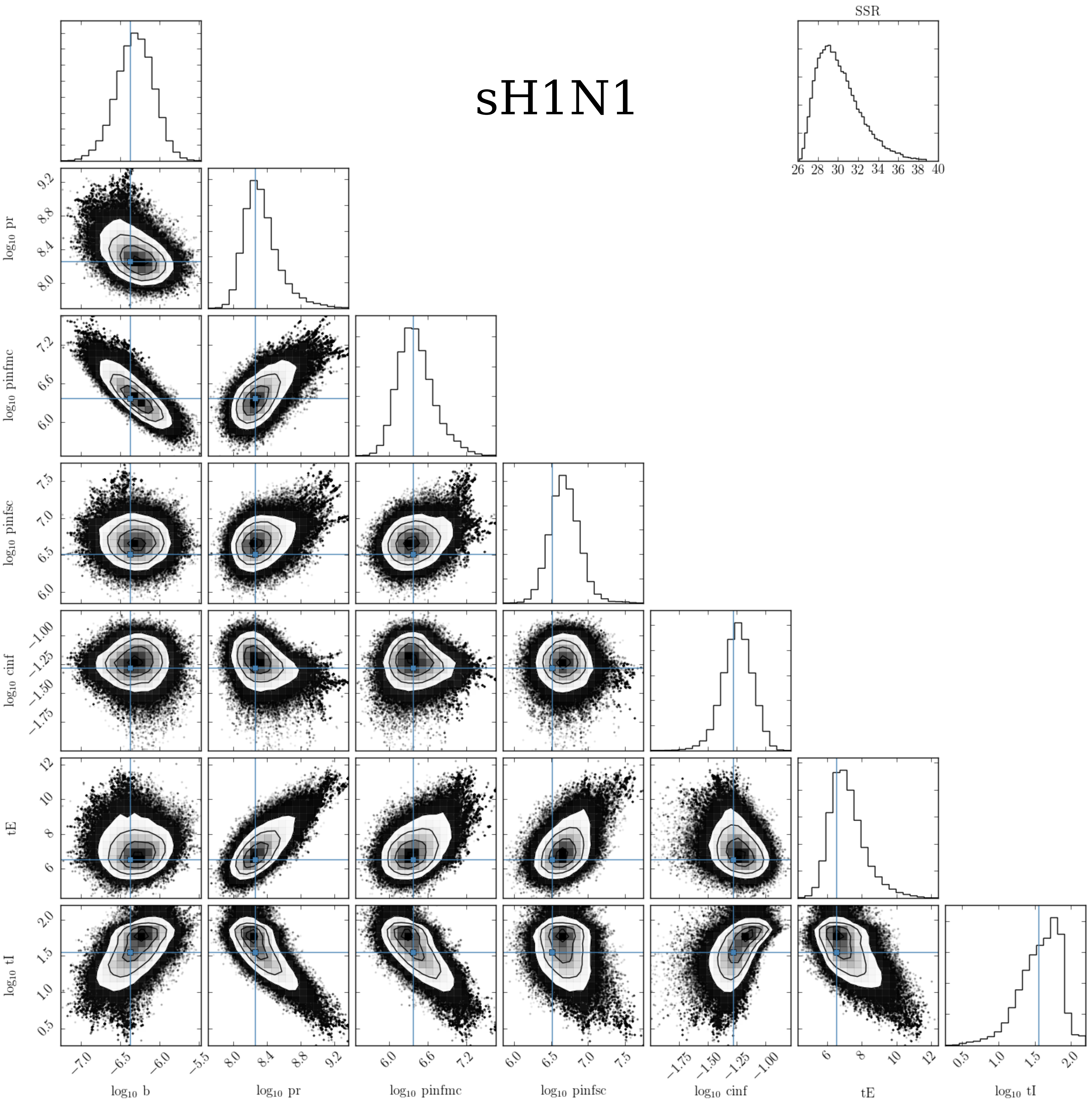
sH1N1
SSR
26 28 30 32 34 36 38 40
log10 b
log10 pr
log10 pinfmc
log10 pinfsc
log10 cinf
tE
log10 tI
−7.0 −6.5 −6.0 −5.5
8.0 8.4 8.8 9.2
6.0 6.6 7.2
6.0 6.5 7.0 7.5
−1.75 −1.50 −1.25 −1.00
6 8 10 12
0.5 1.0 1.5 2.0

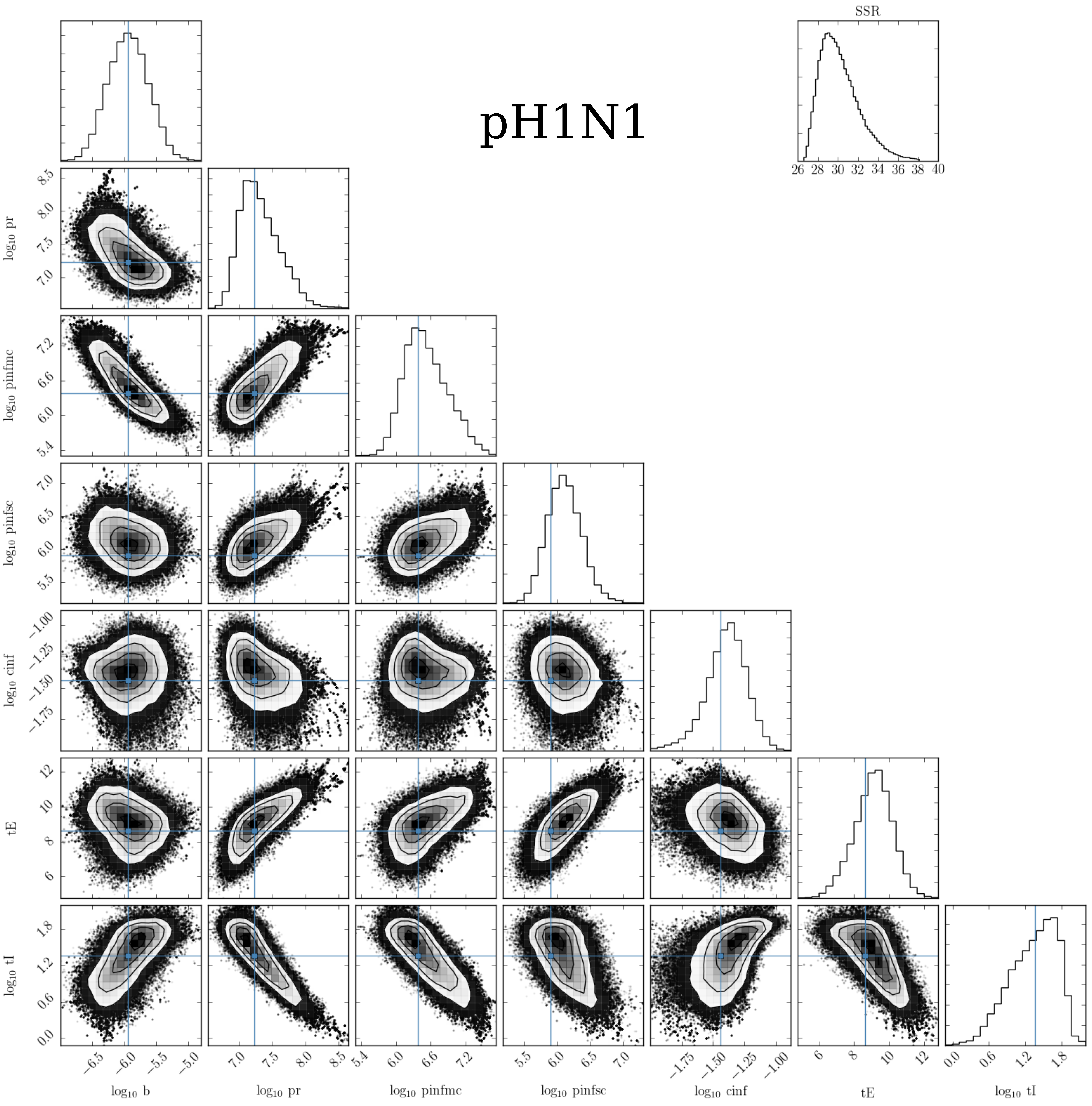

pH1N1
SSR
log10 b
log10 pr
log10 pinfmc
log10 pinfsc
log10 cinf
tE
log10 tI

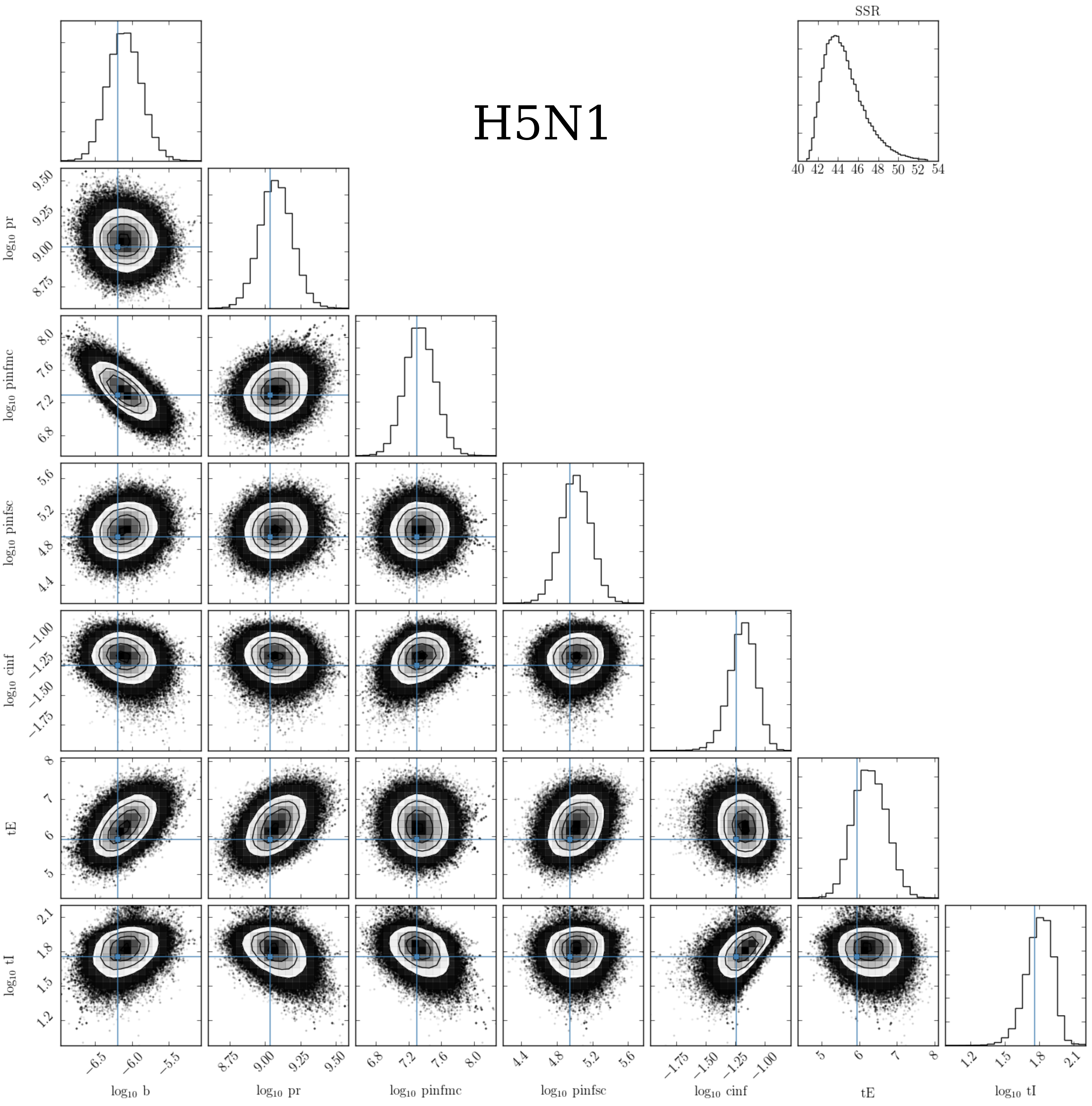

H5N1
SSR
log10 b
log10 pr
log10 pinfmc
log10 pinfsc
log10 cinf
tE
log10 tI

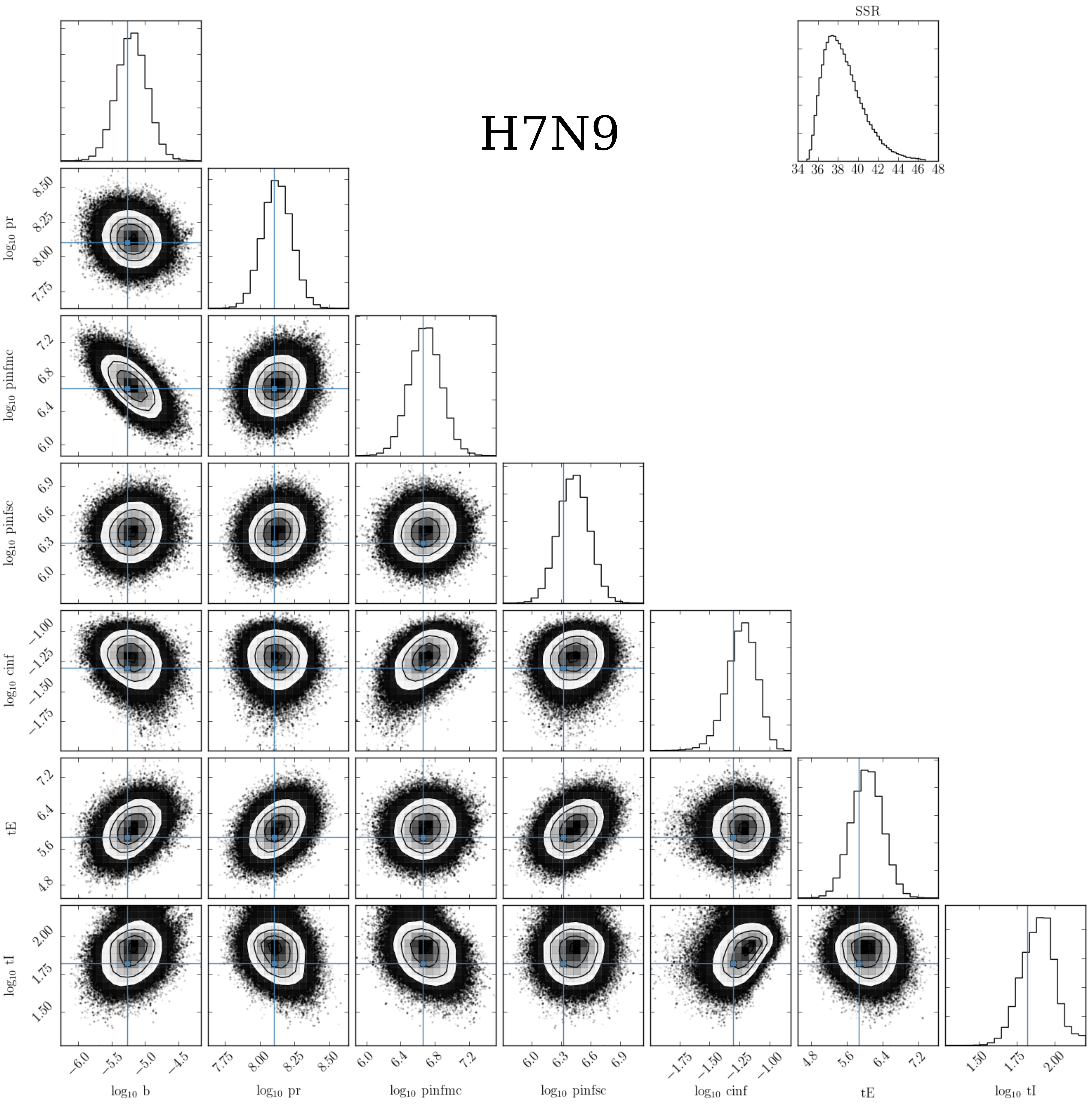
H7N9
SSR
34 36 38 40 42 44 46 48
log10 b
log10 pr
log10 pinfmc
log10 pinfsc
log10 cinf
tE
log10 tI
−6.0 −5.5 −5.0 −4.5
7.75 8.00 8.25 8.50
6.0 6.4 6.8 7.2
6.0 6.3 6.6 6.9
−1.75 −1.50 −1.25 −1.00
4.8 5.6 6.4 7.2
1.50 1.75 2.00
